# Strategic Detection of *Escherichia coli* in the Poultry Industry: Food Safety Challenges, One Health Approaches, and Advances in Biosensor Technologies

**DOI:** 10.3390/bios15070419

**Published:** 2025-07-01

**Authors:** Jacquline Risalvato, Alaa H. Sewid, Shigetoshi Eda, Richard W. Gerhold, Jie Jayne Wu

**Affiliations:** 1 Biomedical and Diagnostic Sciences, College of Veterinary Medicine, The University of Tennessee, Knoxville, TN 37996, USA; rgerhold@utk.edu; 2 School of Natural Resources, The University of Tennessee Institute of Agriculture, Knoxville, TN 37996, USA; asewid@utk.edu (A.H.S.); seda@utk.edu (S.E.); 3 Department of Electrical Engineering and Computer Science, The University of Tennessee, Knoxville, TN 37996, USA; jwu10@tennessee.edu

**Keywords:** *Escherichia coli*, avian pathogenic *E. coli*, poultry diagnostics, biosensors, foodborne pathogens, antimicrobial resistance, point-of-care testing, One Health

## Abstract

*Escherichia coli* (*E. coli*) remains a major concern in poultry production due to its ability to incite foodborne illness and public health crisis, zoonotic potential, and the increasing prevalence of antibiotic-resistant strains. The contamination of poultry products with pathogenic *E. coli*, including avian pathogenic *E. coli* (APEC) and Shiga toxin-producing *E. coli* (STEC), presents risks at multiple stages of the poultry production cycle. The stages affected by *E. coli* range from, but are not limited to, the hatcheries to grow-out operations, slaughterhouses, and retail markets. While traditional detection methods such as culture-based assays and polymerase chain reaction (PCR) are well-established for *E. coli* detection in the food supply chain, their time, cost, and high infrastructure demands limit their suitability for rapid and field-based surveillance—hindering the ability for effective cessation and handling of outbreaks. Biosensors have emerged as powerful diagnostic tools that offer rapid, sensitive, and cost-effective alternatives for *E. coli* detection across various stages of poultry development and processing where detection is needed. This review examines current biosensor technologies designed to detect bacterial biomarkers, toxins, antibiotic resistance genes, and host immune response indicators for *E. coli*. Emphasis is placed on field-deployable and point-of-care (POC) platforms capable of integrating into poultry production environments. In addition to enhancing early pathogen detection, biosensors support antimicrobial resistance monitoring, facilitate integration into Hazard Analysis Critical Control Points (HACCP) systems, and align with the One Health framework by improving both animal and public health outcomes. Their strategic implementation in slaughterhouse quality control and marketplace testing can significantly reduce contamination risk and strengthen traceability in the poultry value chain. As biosensor technology continues to evolve, its application in *E. coli* surveillance is poised to play a transformative role in sustainable poultry production and global food safety.

## 1. Introduction

*Escherichia coli* (*E. coli*) is a gram-negative, rod-shaped bacterium commonly found in the intestines of humans and animals. While most strains are harmless and contribute to intestinal health, certain pathogenic strains can cause severe foodborne illnesses. These pathogenic strains, such as Shiga toxin-producing *E. coli* (STEC), are particularly concerning due to their potential to cause life-threatening conditions like hemolytic uremic syndrome (HUS) [1,2].

*E. coli* is a significant foodborne pathogen, responsible for numerous outbreaks linked to contaminated foods [3]. Contamination can occur in various foods, including leafy greens, sprouts, raw milk and cheeses, and raw beef and poultry [4,5]. The Centers for Disease Control and Prevention (CDC) estimates that foodborne illnesses, including those caused by *E. coli*, affect approximately 48 million people annually in the United States, leading to 128,000 hospitalizations and 3000 deaths. For instance, the CDC reported multiple outbreaks linked to foods such as organic carrots, onions, and raw cheddar cheese in recent years [6].

The poultry industry is particularly vulnerable to *E. coli* contamination, which affects both raw meat products and eggs. Raw poultry meat can harbor pathogenic *E. coli*, posing a risk during slaughter and processing. Effective microbiological sampling and testing are essential to monitor and control contamination in poultry slaughter establishments [7,8]. Additionally, eggs can be contaminated with *E. coli*, especially on the shell surface. Studies have reported that up to 84% of the outer eggshell surface may be contaminated with *E. coli*, making eggs a potential source of foodborne infection [9].

The health implications of *E. coli* infections can range from mild gastrointestinal discomfort to severe, life-threatening conditions. Symptoms typically include abdominal cramps, diarrhea (which can be bloody), vomiting, and, occasionally, fever [10]. In severe cases, particularly with STEC infections, complications such as HUS can arise, leading to acute renal failure and other serious health issues. It is estimated that up to 10% of patients with STEC infection may develop HUS, with a case-fatality rate ranging from 3% to 5% [11].

Intervention measures in food animal production are crucial for reducing the risk of *E. coli* contamination. Pre-harvest management controls and intervention strategies aim to reduce the shedding of pathogenic *E. coli* in cattle, which is a primary reservoir for these bacteria. These measures include [7,8,9] the following:

1. Sanitation Practices: ensuring clean and dry bedding, proper housing, and transportation conditions to minimize contamination.
2. Water and Feed Management: treating drinking water and optimizing feed types and feeding strategies to reduce pathogen load.
3. Direct Anti-Pathogen Strategies: utilizing bacteriophages, competitive exclusion products, and vaccines targeting specific *E. coli* strains.

The One Health approach is integral to addressing food safety issues related to *E. coli*. One Health is a collaborative, multisectoral, and transdisciplinary approach that recognizes the interconnection between people, animals, plants, and their shared environment. By linking human, animal, and environmental health, One Health facilitates comprehensive strategies to prevent and control *E. coli* infections, emphasizing the importance of collaboration across sectors to achieve optimal health outcomes [12].

Detection methods for *E. coli* have evolved significantly, with biosensor-based techniques emerging as a promising approach. Traditional methods like culture-based techniques, enzyme-linked immunosorbent assays (ELISA), and PCR have been complemented by biosensors, which offer rapid, sensitive, and specific detection capabilities [13]. These biosensors utilize various biorecognition elements to identify *E. coli*, providing a valuable tool for enhancing food safety and preventing outbreaks.

This review article delves into the current issues related to food safety concerning *E. coli*, examining its impact, the foods commonly affected, the health implications of infections, and the advancements in detection methods, particularly focusing on biosensor-based techniques.

### 1.1. Current Issues in Food Safety and Poultry Products

Food safety in poultry products is a critical concern due to the potential presence of various pathogens that can cause foodborne illnesses. Among the most significant pathogens are *Salmonella*, *Campylobacter*, and *Listeria monocytogenes*. These bacteria can contaminate poultry during processing and handling, leading to severe health issues if consumed. For instance, *Salmonella* and *Campylobacter* are commonly found in poultry and can cause gastrointestinal illnesses characterized by symptoms such as diarrhea, fever, and abdominal cramps [14]. *Listeria monocytogenes*, although less common, can lead to listeriosis—a serious infection that primarily affects pregnant women, newborns, older adults, and individuals with weakened immune systems [15].

**Figure 1:**
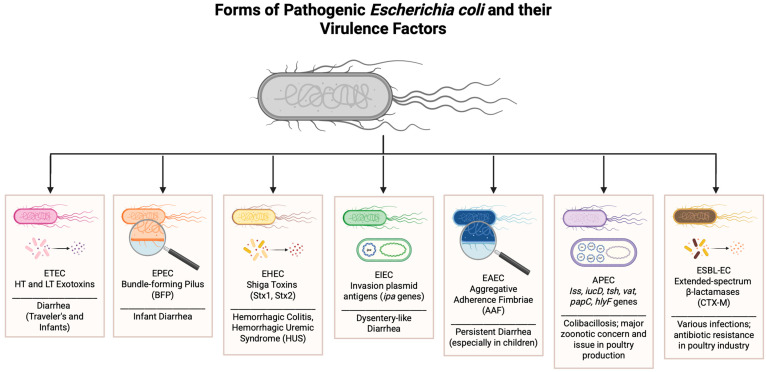
Forms of pathogenic *Escherichia coli* and their Virulence Factors. The virulence factors of ETEC, EPEC, EHEC, EIEC, EAEC, APEC, and ESBL-EC are provided in a visual representation along with their clinical consequence(s). Created in BioRender. Risalvato, J. https://BioRender.com/zsiucc2, (accessed on 2 June 2025).

Among these pathogens, *E. coli* deserves special attention due to its prevalence and impact on poultry products. *E. coli* is a gram-negative bacterium typically found in the intestines of poultry and other animals. While many strains of *E. coli* are harmless, certain strains, known as APEC, can cause colibacillosis, a disease that leads to significant morbidity and mortality in poultry flocks [14]. Colibacillosis can manifest in various forms, including acute septicemia, airsacculitis, pericarditis, and peritonitis [16]. These infections not only affect the health of the birds but also pose a risk to consumers if the bacteria contaminate poultry products [16]. The genomic diversity and pathogenicity of APEC strains are significant, with various serotypes and virulence genes contributing to their ability to cause disease [14]. This diversity complicates efforts to control *E. coli* infections in poultry, necessitating comprehensive strategies for prevention and management [16]. An overview of the strains of E. coli that affect food safety, the animals and humans they affect, and their consequences can be viewed in Figure 1 and Table 1.

The presence of *E. coli* in poultry products is often linked to poor hygienic conditions and inadequate biosecurity measures during production [17]. Contamination can occur at multiple stages, from the farm to the processing plant, highlighting the need for stringent food safety protocols [17]. Effective management practices, such as regular monitoring, proper sanitation, and the use of antibiotics, can help control the spread of *E. coli* in poultry flocks [17]. However, the overuse of antibiotics has led to the emergence of antibiotic-resistant strains, complicating treatment efforts [17]. For instance, extended-spectrum beta-lactamase (ESBL)-producing *E. coli* strains have been identified in poultry, posing a significant challenge due to their resistance to multiple antibiotics [16]. Therefore, preventive measures, including vaccination and improved biosecurity, are crucial in mitigating the risks associated with *E. coli* contamination in poultry products [17]. Research has shown that various disinfection methods can effectively reduce *E. coli* contamination, emphasizing the importance of maintaining high hygiene standards throughout the production process [15].

Ensuring food safety in poultry products requires a comprehensive approach that addresses the various pathogens involved. By emphasizing the control of *E. coli* through improved hygiene and biosecurity measures, the poultry industry can significantly reduce the incidence of foodborne illnesses and enhance consumer safety [18].

### 1.2. Where and When Is E. coli Introduced in the Poultry Production Pipeline

*E. coli* is a common bacterium found in the intestines of poultry and other animals. While many strains are harmless, certain pathogenic strains, such as APEC, can cause significant health issues in poultry, leading to diseases collectively known as colibacillosis [18]. Understanding where and when *E. coli* is introduced in the poultry production pipeline is crucial for developing effective control measures.

#### 1.2.1. Farm Level Introduction

The introduction of *E. coli* in poultry production often begins at the farm level. *E. coli* can be present in the environment, including soil, water, and feed, and can be transmitted to poultry through direct contact or ingestion. Studies have shown that *E. coli* can be found in various environmental samples on poultry farms, including soil, dust, and water sources [19]. The presence of *E. coli* in these environments can be attributed to fecal contamination from infected birds, which can then spread to other birds through contact with contaminated surfaces or ingestion of contaminated feed and water [20].

Poor hygiene practices, such as inadequate cleaning and disinfection of poultry houses, can facilitate the spread of *E. coli* among birds. For instance, the accumulation of fecal matter and litter in poultry houses can create a reservoir for *E. coli*, which can then be aerosolized and inhaled by birds or settle on their feathers and skin [21]. Regular cleaning and disinfection of poultry houses are essential to reduce the bacterial load and prevent the spread of *E. coli* [21]. Additionally, the use of contaminated water for drinking or cleaning can introduce *E. coli* into the poultry population. Water sources can become contaminated with *E. coli* from fecal matter, and birds that drink this water can become infected [16,22]. Ensuring that water sources are clean and regularly tested for contamination is crucial for preventing the spread of *E. coli* [22].

The density of birds in poultry houses also plays a significant role in the spread of *E. coli.* Overcrowded conditions can increase stress among birds, which can weaken their immune systems and make them more susceptible to infections, including those caused by *E. coli* [16,18]. High stocking densities can also facilitate the rapid spread of *E. coli* through direct contact between birds and through the shared environment [16]. Managing stocking densities and providing adequate space for birds can help reduce stress and the risk of *E. coli* infections.

#### 1.2.2. Hatchery and Brooding Stage

The hatchery and brooding stages are critical points where *E. coli* can be introduced into poultry production. Contamination can occur through infected eggs or during the hatching process. *E. coli* can be transmitted from the hen to the egg, and subsequently to the chick, if the eggshells are contaminated [23]. Research has shown that *E. coli* can penetrate the eggshell and infect the embryo, leading to early chick mortality and reduced hatchability [19]. This vertical transmission of *E. coli* from parent to offspring underscores the importance of maintaining strict biosecurity measures in hatcheries.

Hatcheries must implement proper sanitation and handling of eggs and chicks to prevent the spread of *E. coli*. Effective biosecurity measures include regular cleaning and disinfection of incubators, hatchers, and other equipment [20]. Additionally, the use of antimicrobial treatments on eggshells can reduce the risk of *E. coli* contamination [21,23]. Hatchery personnel should follow strict hygiene protocols, such as wearing protective clothing and washing hands frequently, to minimize the risk of spreading *E. coli* [23]. The increased use of gentamycin at the hatchery to control *E. coli* (omphalitis) infection of the umbilical stump has been associated with higher resistance levels during the brooding stage [21].

The brooding stage, where chicks are kept in controlled environments, also requires careful management to prevent *E. coli* infections. Clean bedding, proper ventilation, and regular monitoring are essential to minimize the risk. Studies have shown that contaminated bedding can serve as a source for *E. coli*, which can then infect chicks through contact or inhalation. Proper ventilation helps reduce the concentration of airborne *E. coli* and other pathogens, thereby lowering the risk of respiratory infections [17]. Regular monitoring of chick health and environmental conditions can help identify and address potential issues before they escalate.

The hatchery and brooding stages are critical points where *E. coli* can be introduced into poultry production. Implementing strict biosecurity measures, proper sanitation, and careful management during these stages can significantly reduce the risk of *E. coli* contamination and enhance the overall health and productivity of poultry flocks.

#### 1.2.3. Processing Plant Contamination

The processing plant is another critical point where *E. coli* can be introduced into poultry products. During slaughter and processing, *E. coli* can be transferred from the intestines to the carcass if proper hygiene practices are not followed. Studies have shown that *E. coli* can be present in various parts of the processing plant, including equipment, surfaces, and workers’ hands [22]. Cross-contamination can occur when *E. coli* from the intestines of slaughtered birds contaminates the carcass during evisceration [17]. This contamination can be exacerbated by improper handling and inadequate sanitation protocols.

Effective sanitation protocols, including regular cleaning and disinfection of equipment and facilities, are necessary to prevent *E. coli* contamination. Research indicates that thorough cleaning and disinfection can significantly reduce the bacterial load on surfaces and equipment [19]. For instance, the use of disinfectants such as chlorine-based solutions have been shown to be effective in reducing *E. coli* contamination on processing equipment [18,19]. Additionally, implementing routine sanitation schedules and ensuring that all workers adhere to hygiene practices, such as handwashing and wearing protective clothing, can further minimize the risk of cross-contamination [19].

The implementation of Hazard Analysis and Critical Control Point (HACCP) systems can help identify and control potential contamination points in the processing plant. HACCP is a systematic approach to food safety that involves identifying critical control points (CCPs) where hazards, such as *E. coli* contamination, can be prevented, eliminated, or reduced to safe levels [21,24]. By monitoring these CCPs and implementing corrective actions when necessary, processing plants can effectively manage the risk of *E. coli* contamination [21]. For example, one study demonstrated that HACCP systems could significantly reduce the prevalence of *E. coli* in poultry products by ensuring that critical control measures, such as proper evisceration techniques and effective sanitation protocols, are consistently applied [24,25].

Conclusively, the processing plant is a critical point where *E. coli* can be introduced into poultry products. Effective sanitation protocols and the implementation of HACCP systems are essential to prevent *E. coli* contamination and ensure the safety of poultry products.

#### 1.2.4. Transportation and Storage

Transportation and storage of poultry products present significant opportunities for *E. coli* contamination. During transportation, poultry products can be exposed to various environmental conditions that may facilitate the growth and spread of *E. coli*. For instance, improper handling during loading and unloading can lead to physical damage to the packaging, allowing *E. coli* to contaminate the products [11,19]. Additionally, transport vehicles that are not adequately cleaned and disinfected can serve as reservoirs for *E. coli*, further increasing the risk of contamination.

Inadequate refrigeration during transportation and storage is a critical factor that can promote the growth of *E. coli. E. coli* can multiply rapidly at temperatures above 4 °C, making it essential to maintain proper refrigeration throughout the transportation and storage process [26,27]. Studies have shown that maintaining poultry products at temperatures below 4 °C can significantly reduce the growth rate of *E. coli* and other pathogens [26]. For example, one study demonstrated that *E. coli* counts were significantly lower in poultry products stored at 2 °C compared to those stored at 10 °C [27]. Therefore, ensuring that poultry products are transported and stored at appropriate temperatures is crucial to prevent bacterial growth and ensure food safety.

Packaging materials and methods also play a vital role in minimizing contamination risks. Modified atmosphere packaging (MAP) has been shown to be effective in reducing the growth of *E. coli* and other pathogens in poultry products [17,28]. MAP involves altering the composition of gases within the packaging to inhibit bacterial growth. For instance, packaging poultry products in a mixture of carbon dioxide (CO_2_) and nitrogen (N_2_) can create an environment that is less conducive to the growth of *E. coli*. Research indicates that CO_2_ packaging can significantly reduce the proliferation of *E. coli* compared to traditional air packaging [28]. Additionally, using antimicrobial packaging materials that incorporate substances such as organic acids or silver nanoparticles can further enhance the safety of poultry products by actively inhibiting bacterial growth [28].

Transportation and storage of poultry products are critical points where *E. coli* contamination can occur. Proper handling, adequate refrigeration, and the use of effective packaging materials and methods are essential to minimize the risk of *E. coli* contamination and ensure the safety of poultry products.

#### 1.2.5. Importance of Biosecurity in Poultry Production

Biosecurity is paramount in preventing the introduction and spread of *E. coli* and other pathogens in poultry production. Compliance with biosecurity measures, such as controlling access to poultry houses, using protective clothing, and implementing strict sanitation protocols, can significantly reduce the risk of contamination [26,27,28,29]. For instance, controlling access to poultry houses by restricting entry to essential personnel and using footbaths can minimize the introduction of pathogens from external sources [26]. Protective clothing, including gloves, masks, and coveralls, can prevent the transfer of *E. coli* and other pathogens from workers to poultry [30]. Implementing strict sanitation protocols, such as regular cleaning and disinfection of equipment and facilities, is crucial to maintain a hygienic environment and reduce the bacterial load.

Regular training and education of farm personnel on biosecurity practices are essential to ensure consistent implementation [28]. Studies have shown that continuous education and training programs can significantly improve biosecurity compliance among farm workers [27]. These programs should cover various aspects of biosecurity, including the importance of hygiene, proper handling of poultry, and the use of protective equipment [27]. By educating farm personnel on the latest biosecurity practices and protocols, farms can enhance their ability to prevent the introduction and spread of *E. coli* and other pathogens [26].

Monitoring biosecurity compliance through audits and inspections can help identify potential weaknesses and areas for improvement [31,32]. Audits provide an objective assessment of biosecurity measures and can highlight critical points or areas that require attention [32]. For example, internal audits can evaluate the effectiveness of sanitation protocols, the use of protective clothing, and the control of access to poultry houses [30]. Inspections by external agencies can further ensure that biosecurity standards are met and maintained [31]. Regular audits and inspections can lead to the development of targeted interventions to address identified weaknesses and enhance overall biosecurity [32].

Early, quick, and affordable diagnostic interventions are also essential to maintaining biosecurity and preventing lapses. Rapid diagnostic tools, such as real-time PCR assays, can detect *E. coli* and other pathogens quickly and accurately, allowing for timely intervention [33]. These diagnostic methods can be used to monitor the health status of poultry flocks and identify infections before they spread. Affordable diagnostic technologies, such as deep learning-based disease detection systems, can provide cost-effective solutions for continuous monitoring and early detection. Implementing these diagnostic interventions as part of a comprehensive biosecurity strategy can help maintain the health and productivity of poultry flocks and ensure food safety.

Biosecurity is a crucial step in preventing the introduction and spread of *E. coli* and other pathogens in poultry production. Compliance with biosecurity measures, regular training and education of farm personnel, and monitoring through audits and inspections are essential to minimize the risk of contamination. Early, quick, and affordable diagnostic interventions further enhance biosecurity by enabling timely detection and intervention.

In summary, *E. coli* can be introduced at various stages of the poultry production process, from the farm to processing and storage. Implementing effective control measures, such as stringent biosecurity practices, proper sanitation, and careful handling, is essential to minimize the risk of *E. coli* contamination. By identifying points of introduction and applying comprehensive management strategies and diagnostic detection, the poultry industry can enhance food safety and protect consumer health.

### 1.3. Current Methods of E. coli Detection in Poultry Products

#### 1.3.1. Polymerase Chain Reaction (PCR)

The detection of *E. coli* in poultry products is crucial for ensuring food safety and public health. *E. coli* contamination can lead to severe foodborne illnesses, making accurate and rapid detection methods essential. One of the widely used methods is multiplex PCR, which allows for the simultaneous amplification of multiple target genes. This technique is highly sensitive and specific, making it suitable for detecting *E. coli* in complex samples such as poultry meat.

Multiplex PCR is advantageous because it can identify multiple genes in a single reaction, reducing the time and cost associated with traditional methods. A recent study highlighted the effectiveness of multiplex PCR in identifying *E. coli* by targeting three specific genes: *cydA*, *lacY*, and *ydiV*. These genes are involved in various metabolic processes, making them reliable markers for *E. coli* detection. The study demonstrated high specificity (99.49%) and sensitivity (95.76%), proving to be a reliable and cost-effective tool for rapid *E. coli* detection [34]. The developed multiplex PCR was tested on a large number of Enterobacteriaceae genomes, confirming its high specificity and reproducibility [34].

Another research article evaluated the use of multiplex PCR for diagnosing infections caused by diarrheagenic *E. coli*. The study developed two multiplex PCR assays to detect five categories of diarrheagenic *E. coli* and *Shigella* spp. The assays were directly applied to human diarrheal stool samples, showcasing their potential for rapid and accurate diagnosis [34,35]. This approach can be adapted for use in poultry products to ensure comprehensive monitoring of pathogenic *E. coli* strains.

A further study focused on the application of multiplex PCR in environmental samples, including poultry farms. The researchers developed a multiplex PCR method to detect multiple *E. coli* strains simultaneously. The method was validated using samples from various environmental sources, demonstrating its robustness and applicability in diverse settings [36]. This study highlights the versatility of multiplex PCR in detecting *E. coli* across different matrices, including poultry products.

Multiplex PCR offers several advantages over traditional methods for detecting *E. coli* in poultry products. Traditional methods, such as microbiological culture techniques, involve isolating and culturing bacteria on selective media. While these methods are highly accurate, they are time-consuming, often requiring 24–48 h for bacterial growth and identification [34,37]. Additionally, traditional culture methods may involve multiple steps, including enrichment, isolation, and biochemical testing, which can extend the overall detection timeline [37]. Furthermore, multiplexing and even traditional PCR requires trained personnel that can perform complex steps such as DNA/RNA extraction procedures with minimal cross-contamination, as well as relatively expensive instruments to operate and run these methodologies, and if appropriate genes are not selected, live infections may be missed in the sample.

#### 1.3.2. Antimicrobial Susceptibility Testing (AST)

Another common method for detecting *E. coli* in poultry products is antimicrobial susceptibility testing. This method involves isolating *E. coli* strains from poultry samples and assessing their resistance to various antibiotics. By determining the resistance patterns, this approach not only helps in detecting the presence of *E. coli* but also provides valuable insights into the antimicrobial resistance profiles of the isolates.

A study conducted in Addis Ababa slaughterhouses found a 5.2% prevalence of *E. coli* O157:H7 in poultry meat samples [17]. The antimicrobial susceptibility tests revealed varying levels of resistance among the isolates, with some showing multidrug resistance. This finding is significant as it highlights the potential risk of antibiotic-resistant *E. coli* strains in poultry products, which can pose a threat to public health [17].

Antimicrobial susceptibility testing is essential for monitoring and managing antibiotic use in poultry production. By identifying resistant strains, this method helps in developing targeted strategies to mitigate the spread of resistance. For instance, a review on antimicrobial resistance in bacterial poultry pathogens emphasized the importance of harmonizing testing practices and promoting free access to data on antimicrobial resistance to improve treatment guidelines and monitor the evolution of resistance in poultry bacterial pathogens [38,39]. This approach is crucial for ensuring the effectiveness of antibiotics and preventing the emergence of resistant strains.

Moreover, antimicrobial susceptibility testing provides a comprehensive understanding of the resistance mechanisms and trends over time. A survey of methods used for antimicrobial susceptibility testing in veterinary diagnostic laboratories highlighted the need for standardized practices and technologies to ensure accurate and reliable results [40]. This standardization is vital for comparing data across different studies and regions, ultimately contributing to better management of antibiotic use in poultry production.

Multidrug resistance (MDR) in *E. coli* has significant implications for public health, food safety, and poultry production. MDR *E. coli* strains can cause infections that are difficult to treat due to their resistance to multiple antibiotics. This increases the risk of severe illness and complications, particularly in vulnerable populations such as the elderly, children, and immunocompromised individuals [41]. The transmission of MDR *E. coli* from poultry products to humans can occur through direct contact or consumption of contaminated food, posing a serious threat to public health [42]. Additionally, the presence of MDR *E. coli* in poultry products compromises food safety. Contaminated poultry can serve as a reservoir for resistant bacteria, which can spread through the food chain. This necessitates stringent monitoring and control measures to prevent outbreaks of foodborne illnesses [41,43]. A study in Zhejiang Province, China, highlighted the prevalence of class I integrins in MDR *E. coli* isolates from food-producing animals, emphasizing the role of these genetic elements in the dissemination of antimicrobial resistance genes [43].

MDR *E. coli* can affect not only human health, but poultry health—leading to increased morbidity and mortality rates in flocks. This can result in economic losses for poultry producers due to decreased productivity and higher costs associated with disease management [44]. Effective antimicrobial stewardship and biosecurity measures are essential to mitigate the impact of MDR *E. coli* on poultry production [44]. The emergence of MDR *E. coli* underscores the need for prudent use of antibiotics in poultry farming. The overuse and misuse of antibiotics contribute to the development of resistance, making it crucial to implement guidelines for responsible antibiotic use. Monitoring resistance patterns through antimicrobial susceptibility testing helps in developing targeted strategies to manage and reduce the spread of resistant strains [44]. Lastly, the consequences of MDR *E. coli* can also spread beyond the production chain and into the environment, particularly in areas surrounding poultry farms. Environmental contamination with resistant bacteria can lead to the transfer of resistance genes to other bacterial species, further complicating efforts to control antimicrobial resistance [41]. A study conducted in Nigeria found that MDR *E. coli* isolates were prevalent among poultry workers, chickens, and the poultry farm environment, highlighting the risk of transmission along the food chain [41,43,44].

Despite its utility, AST has several limitations in poultry food safety monitoring. Traditional AST methods are often time-consuming, often requiring at least 24 h for results, which delays rapid intervention during processing. AST also demands specialized lab equipment and infrastructure, as well as highly trained personnel, making this testing modality impractical for field usage in lower-resource settings. Variability in testing protocols can lead to inconsistent results across laboratories and hinder data comparability, and in extreme cases, it can feed into the problem of growing antimicrobial resistance if the wrong pharmaceutical or intervention method is utilized based on incorrect results. Lastly, AST does not distinguish between pathogenic and non-pathogenic strains of *E. coli* or detect specific virulence factors, which limits its diagnostic scope. Through the evolution of resistance mechanisms, some phenotypic tats may miss emerging resistance genes. This highlights the need for complementary genotypic or biosensor-based approaches.

#### 1.3.3. Microbiological Culture

Microbiological culture methods remain a standard practice for *E. coli* detection. These methods involve culturing samples on selective media that support the growth of *E. coli* while inhibiting other bacteria. Selective media, such as MacConkey agar and eosin methylene blue agar (EMB agar), are commonly used to isolate *E. coli* due to their ability to differentiate lactose-fermenting bacteria from non-lactose fermenters [45]. Although traditional culture methods are time-consuming, often requiring 24–48 h for bacterial growth and identification, they are highly accurate and provide detailed information about the bacterial load and characteristics of the isolates [13].

Microbiological culture methods offer several advantages, including the ability to quantify bacterial populations and assess the phenotypic characteristics of the isolates. For instance, a study on bacterial detection and recovery from poultry emphasized the importance of accurate and reproducible methods for quantifying bacterial concentrations in complex samples. Back calculation methods of serial diluted culture at various timepoints can assist in creating growth curves over time and provide a dynamic quantifiable and visual representation of bacteria, including *E. coli*, in both poultry litter and air samples [21]. This approach is essential for monitoring the presence of foodborne pathogens like *E. coli* in poultry products.

Combining culture methods with molecular techniques like polymerase chain reaction (PCR) can enhance the detection accuracy and speed, offering a comprehensive approach to *E. coli* monitoring in poultry products. Molecular techniques, such as multiplex PCR, allow for the rapid and specific identification of *E. coli* by targeting multiple genes simultaneously [34]. This combination provides a robust diagnostic tool, ensuring precise and timely detection of *E. coli* in poultry products [34,35,45].

Microbiological culture methods, while highly accurate, have several limitations when it comes to detecting *E. coli* in poultry products. Firstly, traditional culture methods require significant time for bacterial growth and identification, often taking 24-48 h [45]. This delay can be problematic in situations where rapid detection is crucial for preventing the spread of contamination and ensuring food safety. These methods also involve multiple steps, including sample enrichment, isolation, and biochemical testing, which can be labor-intensive and require skilled personnel [13]. This complexity can limit their practicality in high-throughput settings or smaller laboratories with limited resources.

Culture methods also lack in sensitivity and have limited detection of viable but non-culturable (VBNC) bacteria. Traditional culture methods may fail to detect VBNC *E. coli*, which are alive but do not grow on standard media [13]. These bacteria can still pose a health risk, and their presence may be underestimated using traditional culture techniques. Additionally, while selective media are designed to inhibit the growth of non-target bacteria, they may not be entirely effective in complex samples like poultry products. This can lead to false negatives or the need for additional confirmatory tests [34,35]. Lastly, accurate quantification of bacterial load can be challenging with culture methods, especially in samples with low bacterial concentrations [45]. This limitation can affect the assessment of contamination levels and the effectiveness of intervention strategies.

Combining culture methods with molecular techniques like PCR can help overcome some of these limitations by enhancing detection accuracy and speed [34,35]. For instance, multiplex PCR can rapidly identify multiple target genes, providing a comprehensive approach to *E. coli* monitoring in poultry products [44].

## 2. Biosensor Methods for Detection of *E. coli* in the Poultry Production Pipeline

Biosensors are sophisticated analytical devices that couple bioreceptors with transducers to detect specific biological targets. Through immunological, nucleic acid-based, or and other developing methods, bioreceptors selectively capture analytes and translate biological interactions into measurable signals. The development of robust, highly specific biosensors is critical for the reliable detection of pathogens in complex biological samples, such as blood or fecal material [46,47,48]. Landmark advancements since the early 20th century, including the invention of the hydrogen ion electrode, the oxygen electrode, and PCR, have significantly shaped the field [49,50,51]. Modern biosensors, enhanced by nanotechnology, now offer rapid, non-invasive diagnostic capabilities across medical, environmental, and agricultural applications.

Biosensors employ various transduction methods, including electrochemical, optical, mass-sensitive or piezoelectric transducers, along with key components like bioreceptors and signal processing units, to generate quantifiable outputs [52]. Additionally, biosensors can be classified based on their bioreceptors into categories such as enzymatic, immunosensors, nucleic acid-based sensors, and cellular sensors, each tailored to specific applications. The development of biosensors has benefited from recent progress in nanobiology and nanotechnology, especially with enhanced sensitivity, and the integration of innovative microfluidics with biosensors has promoted the development of portable, cost-effective biosensors for on-site pathogen detection [53]. Notably, recent research has made significant advances in the field through the development of field-deployable biosensors that integrate microfluidic immunoassay platforms and electroanalytical detection modalities [54,55,56]. These innovations have led to the rapid, sensitive detection of pathogens such as *Mycobacterium avium* subspecis. *paratuberculosis* (MAP), demonstrating the potential for early disease detection directly in livestock environments.

In poultry production, early disease detection enabled by biosensors is vital for timely intervention, preventing horizontal transmission, and minimizing economic losses. The success of early intervention depends on identifying progressive biomarkers within a critical detection window that aligns with an opportunity for effective treatment. Integrating biosensors with artificial intelligence platforms and environmental monitoring tools has further enhanced early disease surveillance systems.

Strategies for early detection involve proactive screening during asymptomatic stages or diagnosis based on clinical signs. Biosensors disrupt the “chain of infection” by enabling real-time, point-of-care diagnostics that prevent outbreaks in poultry barns. Enhanced surveillance, combined with smart technologies and continuous monitoring systems, is expected to become increasingly affordable and accessible, offering poultry farmers powerful tools to improve animal welfare, productivity, and disease management.

### 2.1. Types of Biosensors

**Figure 2:**
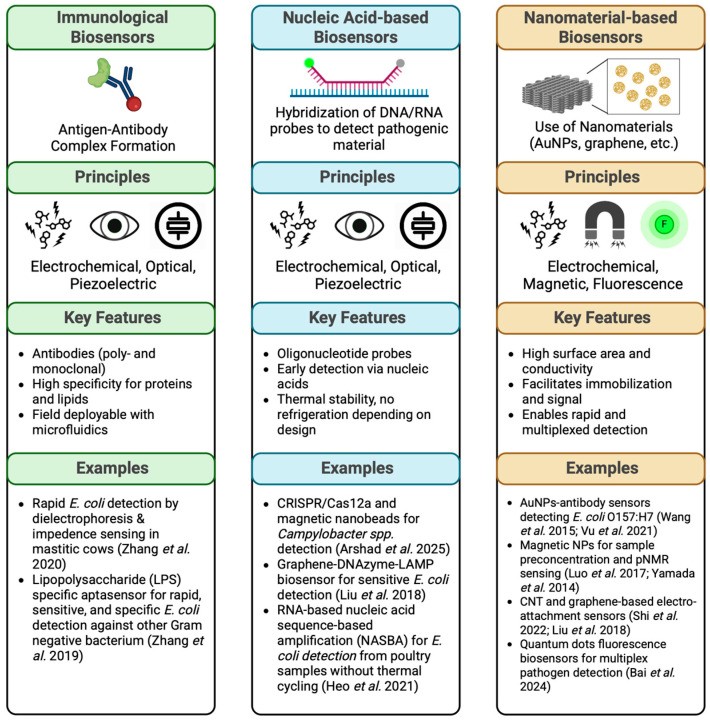
A comparison of immunological biosensors, nucleic acid-based biosensors, and nanomaterial-based biosensors and their principles, key features, and examples of in-the-field use for *Escherichia coli* detection in the poultry industry. References [57,58,59,60,61,62,63,64,65,66,67]. Created in BioRender. Risalvato, J. https://BioRender.com/w4ft2qs, (accessed on 2 June 2025).

In this review, biosensors will be categorized by the agents they detect into the following categories: immunological biosensors, nucleic acid-based biosensors, and nanomaterials of biosensors will be discussed in detail. While there are numerous types of biosensors, for the purposes of this review, our scope and focus will prioritize the detection methods of these platforms. All three of these “types” of biosensors can be used separately or in various combinations with one another, adding to the variety of biosensor development for pathogen detection. Figure 2 shows a depiction of each of these “types” of biosensors and examples of their applications in the poultry industry and research for *E. coli* detection.

#### 2.1.1. Immunological Biosensors

The principle of antigen–antibody complex formation continues to advance diagnostic and biosensor technology. Immunological biosensors are specifically engineered by immobilizing antibodies onto the surface of a transducer, enabling the selective capture of target pathogens with complimentary antigens recognized by the antibodies. Upon the introduction of a sample containing the antigen of interest (in this case, the pathogen of interest), the binding between the antibody and antigen generates a signal such as an electrochemical, optical, or piezoelectric response that can be measured to quantify the pathogen.

In poultry health management, immunological biosensors are critical for the rapid and specific detection of infectious agents. Antibodies are highly effective at recognizing proteinaceous and lipid-based antigens present either intracellularly or on pathogen surfaces, enabling sensitive detection during various stages of infection. Currently, polyclonal antibodies are predominantly utilized due to their broad binding capability and relative ease of production [68,69]. Monoclonal antibodies offer higher specificity compared to polyclonal antibodies; they are often more expensive due to the intensive screen processes and development required for these reagents to have both strong affinity and minimal cross-reactivity.

Work has been underway for the immunological detection of *E. coli* and its detection using biosensors. *E. coli* is not limited to just the poultry industry, but other agricultural industries as well, such as the dairy industry. Beyond antibody-based strategies, the incorporation of nucleic acids, enzymes, and novel microfluidic systems has expanded biosensor versatility. Notably, the work of Jayne Wu and Shigetoshi Eda has significantly advanced the field through the development of field-deployable biosensors that integrate microfluidic immunoassay platforms and electroanalytical detection modalities [59]. Their innovations have led to the rapid, sensitive detection of pathogens such as *Mycobacterium avium* subsp. *paratuberculosis* (MAP), demonstrating the potential for early disease detection directly in livestock environments.

An innovative immunosensor for rapid and quantitative detection of *E. coli* utilizing dielectrophoresis and interfacial capacitance sensing has been developed, through modifications with an *E. coli*-specific antibody on low-cost commercial microelectrodes. This innovative technology achieves a detection limit of 775 cells/mL within 15 s, meeting growing needs for on-site and field diagnostics. Furthermore, speed does not negate specificity with this test, as the sensor had negligible responses to other pathogens such as *Staphylococcus aureus* and *Streptococcus uberis* with a selectivity ratio of 3063:1 with robust validation [57].

These portable devices combine antibody-based capture with label-free impedance measurements, enabling point-of-care diagnostics with minimal sample preparation. Such technological advancements pave the way for improved surveillance, early intervention, and better disease management practices in poultry and broader livestock industries.

#### 2.1.2. Nucleic Acid-Biosensors

Biosensors that utilize the detection of nucleic acid have emerged as essential tools in the game of detecting pathogens, especially those of poultry. Nucleic acid-based biosensors detect the genetic material of pathogens of interest by using oligonucleotide probes based on short sequences of either DNA or RNA. These probes are designed to specifically hybridize with complementary sequences within a pathogen of interest’s genome. When this hybridization occurs, the binding of the oligonucleotide and its probe to the genetic material elicits a signal that can be measured using electrochemical, optical, or piezoelectric transduction methods. These methods allow for highly specific and sensitive detection of target nucleic acid material and pathogen quantification [57,58].

In the poultry industry, nucleic acid-based biosensors have been successfully applied for the rapid identification of a variety of major pathogens, including but not limited to *Campylobacter* spp., *Clostridium* spp., *Salmonella* spp., and *E. coli* [70,71,72,73]. For instance, a microfluidic electrochemical biosensor has been developed to detect *Salmonella* DNA with high specificity in chicken meat samples [74]. Similarly, a CRISPR/Cas12a-based biosensor has been fabricated to identify *Campylobacter* spp. in poultry samples with remarkable sensitivity, leveraging the collateral cleavage activity of Cas12a enzymes [59]. A significant advantage of nucleic acid-based biosensors, including those utilizing aptamers (synthetic nucleic acids with high binding affinities), lies in their thermal stability and robustness under a range of environmental conditions. Unlike antibodies, which often require cold-chain logistics for storage and transport, nucleic acid-based probes can maintain their functionality without refrigeration, making them particularly well-suited for field applications in diverse poultry farming environments [75]. Furthermore, these biosensors excel at detecting infections during the early stages of disease progression, even before clinical signs are evident. Their ability to directly correlate the presence of a pathogen’s genetic material with disease onset enables prompt, on-site diagnosis and intervention, critical for minimizing transmission, ensuring animal welfare, and maximizing economic returns.

Recent technological advancements, such as the integration of biosensors with smartphone-based platforms and portable isothermal amplification techniques like loop-mediated isothermal amplification (LAMP), have further expanded the utility of nucleic acid biosensors in poultry disease surveillance [76,77]. Collectively, these innovations represent a transformative shift toward more accessible, rapid, and reliable disease monitoring systems in the poultry sector, significantly enhancing early detection capabilities and biosecurity measures.

Nucleic acid-based biosensors have additionally greatly improved the detection of *E. coli* in poultry, offering rapid, sensitive, and field-deployable solutions. One innovative strategy involves the use of RNA-cleaving DNAzymes—engineered DNA molecules with catalytic activity—designed to recognize and cleave specific RNA sequences unique to *E. coli*. By integrating DNAzymes with graphene-based materials, a fluorescent biosensor could be created that enables real-time detection of *E. coli* through enhanced fluorescence quenching properties and high sensitivity due to the large surface area and conductivity of graphene [60]. Another strategy involves the use of micro/nanobeads of different materials (magnetic, silica, and polymeric) with various diameters to amplify a quartz crystal microbalance (QCM) sensor for the detection of *E. coli* 0157:H7 [78], exhibiting how different materials can assist with QCM nucleic acid sensing and may be more available as field-deployable options.

A recent study introduced an innovative combination of DNAzyme and LAMP (DNAzyme-LAMP) that offers a promising method for the rapid and sensitive colorimetric and electrochemical detection of general *E. coli* (*phoA* gene) [79] and Shiga toxin-producing *E. coli* strains (*stx1*, *stx2* and *eae* genes) such as O157:H7 [80]. This method successfully detected *E. coli* in milk and vegetable rinse water. Using a novel electrochemical detection approach, the limit of detection reached 6.3 CFU per sample. The entire process can be completed within two hours without the need for expensive equipment, making DNAzyme-LAMP a practical option for rapid and sensitive *E. coli* detection in resource-limited settings such as food processing facilities.

In addition to DNAzyme-based sensors, electrochemical genosensors using gold nanostars have also been developed for *E. coli* O157:H7 detection [81]. These sensors utilize single-stranded DNA probes immobilized onto gold nanostar-modified electrodes that specifically target the *Z3276* gene, a known genetic marker for *E. coli* O157:H7. Upon hybridization of the probe with the target DNA, a quantifiable electrochemical signal is generated, allowing for detection of the pathogen at extremely low concentrations, critical for early-stage identification [81].

Furthermore, RNA-based biosensors have been designed to detect viable *E. coli* cells by targeting specific mRNA sequences. These systems often incorporate isothermal amplification techniques such as nucleic acid sequence-based amplification (NASBA), which amplify RNA targets without the need for thermal cycling, thereby simplifying field deployment [61]. This approach enables the rapid and sensitive detection of *E. coli* directly from complex poultry samples, supporting real-time monitoring and immediate response strategies.

The development and deployment of these nucleic acid-based biosensors in poultry health management are pivotal for early disease detection, minimizing economic losses, improving animal welfare, and enhancing food safety.

#### 2.1.3. The Role of Nanomaterials

By improving sensitivity, specificity, and adaptability for on-site applications, nanomaterials have greatly advanced biosensor technologies for *Escherichia coli* detection in poultry production. Effective immobilization of biorecognition elements and enhanced signal transduction are made possible by their special physicochemical characteristics, which include high surface area-to-volume ratios, electrical conductivity, and biocompatibility.

Gold nanoparticles (AuNPs) have emerged as pivotal components in the development of electrochemical biosensors for the detection of *Escherichia coli* due to their exceptional electrical conductivity, high surface area, and facile functionalization with biomolecules. These properties enable the creation of sensitive and specific detection platforms suitable for various stages of poultry production, particularly during processing where rapid and accurate pathogen detection is critical. For instance, Wang and Alocilja developed a biosensor employing AuNPs conjugated with antibodies to detect *E. coli* O157:H7 in broth samples. This sensor achieved a detection limit of 10 CFU/mL and significantly reduced the detection time to less than one hour, demonstrating its potential for real-time monitoring in food processing environments [62]. In another approach, a label-free electrochemical biosensor utilizing screen-printed electrodes modified with AuNPs demonstrated a detection limit of 15 CFU/mL for *E. coli* O157, with a detection time of approximately 30 min [63]. The integration of AuNPs into biosensor platforms enhances their analytical performance and supports development of portable, field-deployable devices.

Magnetic nanoparticles (MNPs), such as iron oxide-based particles, are instrumental in pre-concentrating bacterial cells from complex poultry matrices. By facilitating magnetic separation, MNPs enhance assay specificity and speed, making them ideal for the farm level and hatchery stages, where early detection can mitigate the spread of pathogens. A study by Luo and Alocilja in 2017 introduced a portable nuclear magnetic resonance (pNMR) biosensor employing antibody-functionalized polymer-coated MNPs for the detection of *E. coli* O157:H7 in complex matrices like milk and water. The biosensor demonstrated detection limits as low as 76 CFU/mL in water and 92 CFU/mL in milk, with results obtainable in approximately one minute, showcasing its suitability for on-site testing in food processing environments [65]. Furthermore, research by Suthindhiran explored the use of magnetosome-based biosensors—biogenic MNPs derived from magnetotactic bacteria—for detecting *E. coli* in contaminated food samples. The magnetosome-antibody complex facilitated the rapid and specific identification of *E. coli* antigens, indicating its applicability in food safety monitoring [82]. Studies in using MNPs in biosensor development have shown that this nanomaterial is effective in rapidly and sensitively detecting *E. coli* in food samples. While current studies have been focused mostly on general food matrices, the principles and methodologies are applicable to poultry production stages where swift detection of contaminants is critical for food safety.

Carbon-based nanomaterials, notably, carbon nanotubes (CNTs) and graphene, have significantly advanced the field of electrochemical biosensors for detecting *Escherichia coli* (*E. coli*), owing to their exceptional electrical conductivity, large surface area, and biocompatibility. These properties enhance the sensitivity and specificity of biosensors, making them particularly advantageous for monitoring *E. coli* contamination during the transportation and storage stages of poultry production, where rapid and accurate detection is crucial to ensure product safety.

CNTs are single-walled carbon nanotubes effectively used in biosensor development—Yamada fabricated a disposable bio-nano combinational junction sensor integrating these single-walled carbon nanotubes and immobilized antibodies for *E. coli* K-12 serotype detection. The sensor demonstrated high sensitivity and specificity, giving potential for CNTs for rapid screening applications in food safety monitoring [64].

Graphene’s unique two-dimensional structure and excellent electrical properties make it an ideal material for biosensor applications. A study by Shi et al. in 2022 [66] developed a graphene aerogel-based biosensing system capable of capturing and detecting *E. coli* with high specificity and sensitivity. The porous structure of the graphene aerogel facilitated efficient bacterial capture, making it suitable for real-time monitoring in food storage environments. Graphene can even be combined with DNAzymes in an innovative approach to create fluorescent biosensors for *E. coli* detection. Li et al. in 2018 reported a graphene-DNAzyme-based biosensor that achieved a detection limit of 10^5^ CFU/mL, demonstrating its applicability for rapid and selective detection of pathogenic bacteria in food samples [60].

Lastly, quantum dots (QDs) are semiconductor nanocrystals renowned for their exceptional fluorescence properties, making them highly effective in optical biosensors for detecting *Escherichia coli* (*E. coli*) and other foodborne pathogens. Their unique optical characteristics, high brightness, and photostability enable the simultaneous detection of multiple pathogens, which is particularly advantageous during the marketplace quality control stage of poultry products. One notable application involves the use of semiconductor QDs as fluorescent labels in immunosensors for the simultaneous detection of *E. coli* O157:H7, Salmonella Typhimurium, and Listeria monocytogenes. In this approach, QDs with distinct emission wavelengths (525 nm, 610 nm, and 705 nm) were conjugated to specific antibodies targeting each pathogen. The pathogens were first separated from food samples using antibody-coated magnetic microbeads. The bead–pathogen complexes were reacted with the QD–antibody conjugates to form bead–pathogen–QD complexes. Fluorescence emission peaks corresponding to each pathogen were measured, achieving detection limits as low as 10^4^ CFU/mL within two hours. This method demonstrated high specificity and sensitivity, underscoring its potential for rapid screening in food safety applications [62].

Another innovative strategy combines QDs with aptamers and silver nanoparticles to construct a fluorescence resonance energy transfer (FRET)-based biosensor. In this system, carbon quantum dots (CQDs) synthesized from grapefruit peel served as the energy donor, while silver nanoparticles acted as the energy acceptor. The presence of *E. coli* disrupted the FRET process, leading to a measurable fluorescence change. This biosensor achieved a detection limit of 10 CFU/mL, highlighting its efficacy for the sensitive detection of *E. coli* in food samples. These advancements in QD-based biosensors offer significant benefits for the poultry industry, particularly at the marketplace quality control stage [67]. The ability to rapidly and accurately detect multiple pathogens ensures that contaminated products are identified before reaching consumers, thereby enhancing food safety and public health outcomes.

### 2.2. Screening for Detection

#### 2.2.1. Life Stages

The detection of *E. coli*, particularly APEC strains, remains critical throughout poultry production due to their association with systemic infections, increased mortality, and economic loss. Advanced biosensor technologies capable of detecting nucleic acids and bacterial antigens have emerged as valuable tools for early intervention, spanning the embryonic (in ovo) stage through to grow-out and pre-harvest stages.

#### 2.2.2. In Ovo

In ovo biosensing approaches aim to identify infections before hatching, limiting vertical transmission and improving hatchery sanitation. When testing eggs in the production line, embryonic fluids (e.g., allantoic fluid) are collected for diagnostic testing by carefully aspirating the fluid using a sterile needle from a small opening in the eggshell under sterile conditions. In some cases, this sampling is guided by microscopy to avoid harming the embryo if the egg is fertilized and intended for hatch. Additionally, testing can take place via swabs on the outside of the eggshell, which causes no harm to the embryo or product.

Microfluidic-based nucleic acid biosensors have demonstrated the ability to detect bacterial DNA in embryonic fluids with high sensitivity. While not a bacterium, infectious bronchitis virus (IBV) DNA was able to be detected to a limit of detection of 2 × 10^−12^ and 10^−5^ mol/L using an electrochemical DNA biosensor containing a multi-walled CNT-modified gold electrode on embryonic fluids from eggs—showing promise in the detectability of nucleic acids of other pathogens in egg products [83]. *E. coli* O157:H7 has been detected in yogurt and eggs using a field-portable fluorescent imager on a smartphone device with a compact laser-diode photosource and a sandwich ELISA assay at a limit of detection of 1 and 10 CFU/mL, respectively [84]. These biosensor examples provide a minimally invasive method for pathogen detection, helping hatcheries take early corrective actions without sacrificing viable embryos.

#### 2.2.3. Hatchlings

Newly hatched chicks are particularly vulnerable to *E. coli* colonization through environmental exposure or suboptimal biosecurity practices. Biosensor systems that can offer rapid screening options for identifying *E. coli* immediately post-hatch can provide a valuable tool for early intervention in poultry production. A microfluidic biosensor was developed by Muhsin et al., 2022 for the detection of *E. coli* 0157:H7 in tap water and wastewater—both providing a viable field-deployable option for *E. coli* detection in the environment regarding water sources as a potential source of contamination and wastewater as a testing source to monitor hatchling health [85]. Early identification allows for immediate interventions like targeted antibiotic therapy or probiotic administration to mitigate early morbidity.

#### 2.2.4. Monitoring During Growth and Maturation

As poultry mature, exposure to *E. coli* through contaminated litter, feed, or water remains a constant risk. Newly hatched chicks are highly susceptible to the vertical and horizontal transmission of *E. coli*, and though antibiotic washes of eggs pre-hatch are a standardized practice, this regular use of antibiotics has been associated with increases in AMR bacteria [86]. Kumar et al., 2025 developed a label-free capacitive biosensor utilizing interlocked Prussian Blue and graphene oxide networks on a screen-printed carbon electrode capable of detecting H5N1 (avian influenza) nucleic acid and the capsular proteins of *E. coli* [87]. The ability of this biosensor to be used on aerosols opens the door for consistent monitoring during much of the grow-out phase and during turnover in the poultry house post-harvest before reintroduction of new animals. Additionally, field-deployable CRISPR-Cas-based biosensors provide a highly sensitive, amplification-free method for detecting *E. coli* DNA directly from environmental and bird samples [88]. These biosensors enable the regular monitoring of flock health and support early management decisions to prevent large-scale outbreaks.

The integration of these biosensor technologies throughout the life cycle of poultry production not only reduces reliance on laboratory-based diagnostics but also enables continuous, real-time monitoring. Such surveillance systems, when combined with integrative software and systems such as the Internet of Things (IoT) networks, can drastically improve biosecurity, animal welfare, and productivity outcomes. Table 2 provides an overview of biosensor screening developments over different life stages of poultry development and growth.

### 2.3. Markers of Interest

#### 2.3.1. Antibiotic Screening and Resistance

Ensuring poultry products are free from antibiotic residues is critical for consumer safety and public health. Regulatory frameworks mandate appropriate withdrawal periods after antibiotic administration to prevent residues in meat and eggs [89]. Despite these measures, residues of antibiotics such as tetracyclines, β-lactams, quinolones, and aminoglycosides are still frequently detected in poultry products, including muscle tissues, livers, giblets, and eggs [90,91,92]. Historically, the sub-therapeutic use of antibiotics for growth promotion has contributed to widespread misuse, leading to an alarming increase in antimicrobial resistance (AMR) [93]. Although regulatory efforts, including the FDA’s 2022 report citing a 70% reduction in antimicrobial sales for chickens since 2016 [94], have helped curb antibiotic use in poultry grow-out facilities, antibiotic exposure during hatchery processes, such as egg dipping before incubation, remains a concern for early resistance development in pathogens like *E. coli*.

Monitoring AMR in *E. coli* across the poultry life cycle, from in ovo stages to mature broilers, is essential for early intervention. Conventional detection methods, such as PCR assays and metagenomics, offer detailed insights into resistance genes, yet these techniques are laborious, require sophisticated laboratory infrastructure, and are unsuitable for rapid, field-based screening [95,96]. Similarly, minimum inhibitory concentration (MIC) assays remain the standard for determining antimicrobial susceptibility but are time-consuming and impractical for large-scale, on-site testing [97]. In response to the detection of antimicrobial-resistant *Escherichia coli*, interventions such as vaccination, disinfection protocols, and rigorous sanitation measures are routinely implemented. These strategies aim to prevent the entry of contaminated products into the food supply chain, eradicate the pathogen from the production environment, and mitigate its dissemination within broiler or layer facilities, which are potential sources of origin.

Biosensor technologies have emerged as promising alternatives for the rapid detection of *E. coli* and its antibiotic resistance profiles in poultry. For instance, Sun et al. (2020) [98] developed a colorimetric and electrochemical bioassay utilizing p-benzoquinone as a redox mediator, capable of detecting *E. coli* via visible colorimetric changes—even colorimetric changes that are difficult to detect due to trimethoprim-resistance that affects the redox reactions of p-benzoquinones—captured by smartphones. The developed colorimetric assay quantified the minimum color change associated with both low and high trimethoprim-resistant *E. coli* bacterium, showing high specificity and sensitivity. This portable system enables field-friendly, quantitative monitoring of bacterial loads and resistance profiles. Furthermore, graphene-assisted biosensors have been developed to detect the AMR of *E. coli* by mixing the bacteria with various antibiotics and measuring the reduced electrocatalytic reduction signal, or the lack thereof if AMR to that antibiotic was present [99,100].

Targeting *E. coli* at multiple production stages can significantly improve food safety. In ovo detection using electrochemical biosensors would do so for the early identification of pathogens before hatch, preventing the vertical transmission of resistant strains—and there is growing promise with current in ovo pathogen detection strategies by biosensors for future AMR detection development [83,84]. Post-hatch, during grow-out, integrated biosensors in feed systems and barn environments can monitor pathogen loads and alert to AMR emergence, enhancing biosecurity protocols [101]. Mehlhorn et al. reviews the potential for aptamer-based biosensors for antibiotic detection and their multiplexed capabilities for a variety of antibiotics used in the poultry industry, primarily gentamicins, tobramicins, and sulfonamides—which are specifically used in the poultry and other farmed animal industries to combat *E. coli* [102]. Combined with the technology of Kumar et al.’s Prussian Blue and graphene oxide biosensor network for *E. coli* detection in aerosolized samples, multiplexation is possible for the presence of both antibiotics and bacteria—which would elucidate the presence of antibiotic resistance [87]. It would also be possible for future development into detection of antibiotic resistance genes in these aerosolized samples as well.

Lastly, *E. coli* can be detected in meat post-slaughter—Helali et al. detected *E. coli* using an electrochemical impedance biosensor with an anti-*E. coli* antibody for the K12 strain and was detectable even in poultry meat products frozen for 45 days [103]. While not specifically used for AMR detection, the ability to test meat products post-production allows pathways for the development of possible future methods of AMR detection for poultry products that make it from slaughter to grocery store shelves.

The integration of biosensors into poultry production chains offers a proactive strategy to mitigate AMR risks, as further shown in Table 3. Future platforms, particularly those based on aptamer arrays and nanomaterials, are likely to support multiplexed, real-time screening, directly informing antibiotic stewardship and disease management practices in poultry farming.

#### 2.3.2. Bacterial Toxins

*E. coli* represents a major pathogen in poultry, not only due to direct bacterial infection but also because of the production of potent bacterial toxins that exacerbate disease severity and impact poultry health. Among these, Shiga-like toxins (Stx), heat-labile enterotoxins (LT), and heat-stable enterotoxins (ST) are of particular concern due to their roles in gastrointestinal and systemic diseases [104]. Early detection of these toxins in poultry production environments is critical for preventing disease outbreaks, ensuring animal welfare, and safeguarding public health.

Traditional methods for toxin detection, such as ELISA and PCR-based assays, while highly specific, are often time-consuming, require laboratory infrastructure, and may not be practical for real-time, on-site monitoring. ELISA, as an immunoassay, can utilize antibodies to specific toxin proteins to detect active toxin production, whereas PCR modalities are capable of sensitively detecting the presence of toxin genes (DNA/RNA) and the genetic potential to produce a toxin—but not, technically, the presence of a toxin in a live culture [105]. Consequently, biosensor technologies have been increasingly explored as rapid, sensitive, and field-deployable alternatives for detecting *E. coli* toxins directly from poultry samples, such as feces, drinking water, or processing surfaces.

Electrochemical biosensors have shown promise in this field. For instance, one method is an aptamer-based electrochemical biosensor capable of detecting *Stx2* with high sensitivity using gold nanoparticle-modified electrodes [106]. This approach allows real-time monitoring of toxin presence without the need for extensive sample preprocessing, which is critical in poultry barns or processing facilities. Similarly, optical biosensors employing surface plasmon resonance (SPR) have been utilized to detect heat-labile enterotoxins by immobilizing specific antibodies or aptamers on sensor chips, enabling multiplexed toxin detection with minimal turnaround time [107].

Recent developments in label-free biosensors, particularly those based on surface-enhanced Raman spectroscopy (SERS), have further enhanced the capabilities for detecting bacterial toxins. A study by Koya et al., 2019 demonstrated that Raman spectroscopy, without the need for enzymatic immunoassays, provides moderate-to-high sensitivity and low-to-moderate specificity and overall better performance than traditional toxin enzyme immunoassays for the detection of *Clostridium difficile* toxins TcdA and TcdB in human stool, suggesting its applicability in low-contamination settings typical of commercial poultry farms for the detection of other bacterial entero- and exotoxins [108].

The integration of these biosensor platforms into poultry production can allow continuous surveillance of environmental reservoirs of *E. coli* toxins, such as litter, feed, and water systems. Portable devices equipped with toxin biosensors could be deployed for routine monitoring, identifying contamination hotspots before large-scale outbreaks occur. Moreover, combining toxin detection with pathogen identification (e.g., multiplex biosensors) would provide a comprehensive assessment of both bacterial presence and virulence potential, enhancing biosecurity measures throughout the poultry production cycle.

As antimicrobial resistance continues to limit treatment options for bacterial infections, the early detection and control of toxin-producing *E. coli* strains using biosensor technology will become an increasingly critical component of sustainable poultry health management.

#### 2.3.3. Biomarkers and Immune Responses

In poultry production, the rapid and accurate detection of *E. coli* infections is critical for maintaining flock health and preventing large-scale outbreaks. Beyond direct pathogen detection, monitoring biomarkers associated with bacterial presence and the host’s immune response can offer a more comprehensive understanding of infection dynamics. Biosensors have emerged as promising tools for real-time, sensitive, and on-site detection of both bacterial biomarkers—such as lipopolysaccharides (LPS) and virulence factors—and host immune indicators, including cytokines and acute-phase proteins.

Electrochemical biosensors have been widely investigated for detecting LPS, a major outer membrane component of *E. coli* and a potent endotoxin responsible for triggering host inflammatory responses. For example, a highly sensitive electrochemical aptasensor capable of detecting ultra-low concentrations of LPS was developed by Zhang et al., 2018, which enabled early-stage infection surveillance in poultry environments [58]. Similarly, colorimetric biosensors using gold nanoparticles functionalized with LPS-specific aptamers have been proposed as simple, field-deployable solutions for LPS monitoring in poultry litter and drinking water [109].

In addition to pathogen-derived biomarkers, monitoring host immune responses provides valuable information regarding disease progression and the efficacy of interventions. Biosensors designed to detect chicken cytokines and acute-phase proteins would be beneficial in diagnostic applications for early infection detection and help mitigate outbreak impact. Notably, a multiplexed immunoassay for chicken cytokines has been developed by Zhong et al., 2019, allowing for detection via a chemiluminescence chip bioarray of chicken interleukin-4 (IL-4) and interferon-gamma (IFN-γ). This research offers a potential tool and buildable platform for evaluating early immune activation in response to bacterial infections [110].

The integration of dual-target biosensors capable of simultaneously detecting both bacterial antigens and host immune markers holds significant promise. For instance, electrochemical immunosensors have been proposed for multiplex detection, allowing the concurrent measurement of *E. coli* biomarkers and host-derived inflammatory proteins in poultry blood or serum samples [111]. Such biosensor platforms would enable veterinarians and producers to differentiate between mere colonization and active systemic infection, informing more targeted management strategies.

Furthermore, the use of label-free biosensing techniques such as surface-enhanced Raman spectroscopy (SERS) and impedance spectroscopy offers the possibility of real-time monitoring without the need for complex sample preparation [108]. These advancements, combined with the miniaturization of biosensor devices, could revolutionize poultry disease surveillance by offering cost-effective, sensitive, and rapid diagnostic tools applicable throughout different stages of poultry production.

As antibiotic resistance rises and concerns over food safety intensify, deploying biosensors that can detect *E. coli* infections through both pathogen and host immune signals will be essential for sustainable poultry farming practices.

#### 2.3.4. Zoonotic Potential

*E. coli*, a common commensal of the avian gut, can become pathogenic, posing significant zoonotic risks to human health through contaminated poultry products. Certain strains, such as APEC, are particularly concerning due to their genetic similarities to extraintestinal pathogenic *E. coli* (ExPEC) strains implicated in human urinary tract infections and sepsis [112]. The early and accurate detection of zoonotic *E. coli* strains at multiple points within the poultry production cycle is, therefore, critical to improving food safety and mitigating public health risks. Biosensors have emerged as rapid, sensitive, and field-deployable alternatives to conventional microbial culture and PCR methods for monitoring these pathogens.

Biosensors targeting specific virulence genes, such as *iss* (increased serum survival), *iutA* (iron uptake) and *hlyF* (hemolysin F), which are commonly associated with zoonotic APEC strains for use in the poultry environment, would be a tremendous benefit to the industry and a much-needed niche to fulfill in biosensor development. As not all *E. coli* strains are pathogenic, looking specifically for virulence genes can assist in assessing the level of biocontainment required and alleviate financial burdens on the producers. For example, a graphene-based electrochemical DNA biosensor demonstrated high specificity in detecting *E. coli* DNA sequences directly from environmental samples such litter and tap and wastewater [85,87]. These platforms provide a significant advantage over culture-based techniques by drastically reducing the time to result—from days to just a few hours—thus enabling more proactive management of foodborne risks.

Label-free biosensors utilizing surface-enhanced Raman spectroscopy (SERS) have also been applied for the identification of zoonotic *E. coli* strains. SERS platforms functionalized with *E. coli*-specific aptamers or antibodies can detect pathogens at very low concentrations, even in complex matrices like raw poultry carcasses [113]. Recently, the development of multiplex biosensors has allowed for the simultaneous detection of multiple zoonotic bacteria, including *E. coli* and *Salmonella* spp., using microfluidic chips coupled with fluorescent or electrochemical readouts [114]. Such multiplex systems are particularly valuable at poultry processing plants where contamination with multiple zoonotic agents often occurs.

Importantly, biosensors are now being integrated with portable detection systems, such as smartphone-based readers, allowing real-time pathogen monitoring across the farm-to-fork continuum. Yang et al. developed a smartphone-interfaced colorimetric biosensor capable of detecting *E. coli* O157:H7 directly from chicken meat, which offers itself as a practical tool for both producers and food inspectors [115].

The deployment of biosensors in poultry production not only improves the early detection of zoonotic *E. coli* but also supports the implementation of targeted interventions, reducing the risk of contaminated products entering the human food chain, as overviewed in Table 4. Continued advancements in nanomaterials, microfluidics, and biomolecular engineering are expected to further enhance biosensor performance, making them indispensable tools in modern poultry health management and zoonotic disease prevention.

### 2.4. Strategic Implementation

The strategic application of biosensors for the detection of *E. coli* represents a critical advancement in poultry industry biosecurity and food safety. As poultry products are a common vector for zoonotic transmission of pathogenic *E. coli* strains, particularly APEC and enterohemorrhagic *E. coli* O157:H7, early and effective detection strategies are essential. Biosensors, offering rapid, sensitive, and on-site detection capabilities, are positioned as valuable tools to complement and enhance traditional microbiological methods. Two crucial stages in the poultry production chain—slaughterhouse processing and marketplace distribution—present significant opportunities for strategic biosensor deployment.

#### 2.4.1. Slaughterhouse Quality Control

Slaughterhouses are primary intervention points where *E. coli* contamination can occur, notably, during defeathering, evisceration, and chilling processes [116,117]. Integrating biosensor technologies at these critical control points (CCPs) allows for the immediate detection of bacterial contamination and minimizes the risk of downstream contamination. Electrochemical biosensors, particularly those utilizing graphene-based or gold nanoparticle-modified electrodes, have shown exceptional promise. These platforms have demonstrated the capacity to detect pathogenic *E. coli* strains at concentrations as low as 10^2^ CFU/mL within carcass rinse and environmental samples [85,87,113,118].

Surface-enhanced Raman spectroscopy (SERS) biosensors, combined with aptamer-based capture systems, provide additional advantages through high sensitivity and the potential for multiplex detection of multiple pathogens simultaneously [113]. Biosensor integration at slaughter facilities enables rapid screening post-evisceration or pre-chill, informing immediate interventions such as reprocessing, targeted sanitation, or carcass segregation. This strategic application enhances compliance with food safety regulations, reduces recalls, and supports the implementation of HACCP plans tailored to microbial monitoring.

#### 2.4.2. Marketplace Quality Control

Beyond processing facilities, the need for pathogen monitoring extends into retail and marketplace environments where poultry products are directly accessible to consumers. Here, the strategic deployment of portable, user-friendly biosensor systems provides a second line of defense against foodborne illness outbreaks. Smartphone-based colorimetric biosensors, capable of detecting *E. coli* O157:H7 contamination within 30 to 60 min, offer a low-cost and accessible approach for use in smaller retail outlets and regulatory inspections [113,119].

Additionally, microfluidic lab-on-a-chip platforms have been developed for rapid, multiplexed pathogen detection directly from meat samples [120]. These systems require minimal operator training and can produce reliable results at the point of sale, thus enhancing the transparency and safety of the poultry supply chain. The marketplace implementation of biosensors not only ensures regulatory compliance but also promotes consumer confidence through improved product traceability and safety assurances.

Strategically embedding biosensor systems into slaughterhouse and marketplace workflows strengthens the poultry industry’s ability to manage microbial risks proactively. By enabling the rapid detection of zoonotic *E. coli* strains at multiple points in the production and distribution chain, these technologies play a pivotal role in safeguarding public health and maintaining the economic integrity of poultry production systems [94,121].

### 2.5. Benefits

The implementation of biosensor technologies for the detection of *E. coli* in the poultry industry offers a transformative opportunity to improve food safety, enhance production efficiency, and protect public health. Traditional microbiological methods, such as culture-based techniques and PCR, though reliable, are often labor-intensive and time-consuming, and they require centralized laboratory infrastructure [122]. In contrast, biosensors provide a platform for rapid, sensitive, and cost-effective detection that can be deployed directly at critical points within the poultry production and distribution chains. Among the many advantages biosensors offer, three core benefits—affordability, time efficiency, and ease of use at the point of care—position them as an essential technology for the future of poultry biosecurity.

#### 2.5.1. Affordability

Affordability remains a primary driver for the widespread adoption of biosensors in poultry production, especially for small- to medium-scale operations. Biosensors based on electrochemical, colorimetric, and immunoassay platforms have been developed with materials such as graphene, gold nanoparticles, and aptamers, which significantly reduce production costs compared to conventional laboratory equipment [123,124]. Portable devices integrating low-cost fabrication methods, including screen-printing and microfluidic chip technologies, enable on-site *E. coli* detection with minimal investment [120]. The cost savings realized from early detection—preventing the need for broad recalls or culling due to undetected outbreaks—further underscore the economic value of integrating biosensors into poultry safety management systems.

#### 2.5.2. Time

Speed is critical in pathogen surveillance to prevent widespread contamination events. Traditional methods may require up to 24–72 h to confirm the presence of *E. coli*, whereas biosensors offer results within minutes to a few hours [125]. Recent developments in electrochemical impedance biosensors and smartphone-assisted colorimetric assays have enabled *E. coli* detection with high sensitivity (as low as 10^2^ CFU/mL) in under one hour [114,123]. Rapid detection enables timely interventions such as carcass segregation, enhanced sanitation, and antimicrobial treatments, thus reducing the risk of downstream contamination in both slaughterhouses and markets. Speedier testing also helps regulatory authorities and producers comply with increasingly stringent food safety regulations without impeding workflow.

#### 2.5.3. Ease of Use

One of the most significant advantages of biosensor platforms is their adaptability for use as point-of-care (POC) tests in non-laboratory settings. Many biosensors designed for *E. coli* detection require minimal technical training and no specialized laboratory infrastructure [120,123]. Paper-based biosensors, smartphone-integrated detection platforms, and lab-on-a-chip systems are examples of technologies that allow frontline workers, veterinarians, and regulatory inspectors to perform accurate diagnostic tests directly at farms, processing facilities, or markets. Such ease of use promotes more frequent surveillance, democratizes access to food safety monitoring tools, and strengthens the industry’s ability to rapidly respond to potential zoonotic threats.

**Figure 3:**
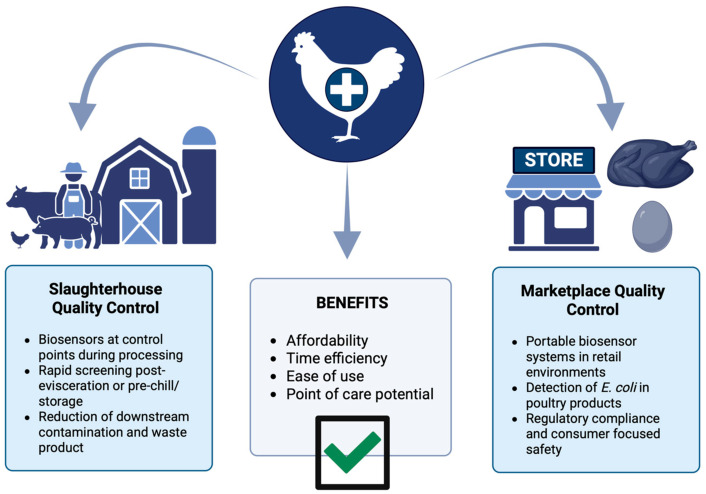
How strategic implementation of biosensors for foodborne pathogen detection in poultry slaughterhouse and marketplace quality control can lead to improved food safety benefits. Created in BioRender. Risalvato, J. https://BioRender.com/rxnvvd0, (accessed on 2 June 2025).

Together, these advantages—affordability, rapid turnaround time, and ease of use—highlight the critical role of biosensors in modernizing poultry safety protocols. By strategically integrating these technologies across different points in the production and distribution chain, the poultry industry can significantly enhance its capacity to control *E. coli* contamination, safeguard consumer health, and improve the economic resilience of poultry operations. Figure 3 displays how strategic implementation in the slaughterhouse and marketplace quality control chain can reap the benefits of biosensors for *E. coli* and foodborne pathogen detection in general.

## 3. Conclusions

The detection and control of *E. coli* in the poultry industry remains a critical challenge for global food safety, public health, and animal welfare. While most *E. coli* strains are commensal, pathogenic variants—including Shiga toxin-producing and avian pathogenic strains—pose significant risks due to their zoonotic potential and ability to cause severe illnesses in humans. As poultry products continue to be identified as frequent vectors in foodborne outbreaks, there is a clear need for robust, sensitive, and field-deployable detection systems that can operate across various stages of production, from hatcheries and grow-out barns to slaughterhouses and retail markets.

Traditional diagnostic tools, such as culture-based assays, ELISA, and PCR, remain valuable but are often hindered by limitations in speed, cost, and accessibility, particularly in decentralized or low-resource settings. In this context, biosensor technologies have emerged as powerful alternatives that offer the rapid, sensitive, and cost-effective detection of *E. coli* and its associated virulence factors, toxins, and resistance markers. Immunological biosensors, nucleic acid-based platforms, and next-generation aptamer- and nanomaterial-enabled systems provide the flexibility to monitor both the pathogen and host responses in real time, opening new pathways for early diagnosis, intervention, and surveillance.

The strategic implementation of biosensors within poultry production systems offers multifaceted benefits. At the slaughterhouse level, biosensors can serve as critical control tools, allowing processors to detect contamination during key processing stages and to make rapid, evidence-based decisions to reduce carcass rejection and prevent downstream contamination. In marketplaces, portable biosensor platforms enhance consumer safety and confidence by enabling last-mile verification of product quality and microbiological safety. These technologies align with modern food safety frameworks and international regulatory expectations, particularly when integrated into HACCP-based protocols.

Moreover, biosensors play an essential role in supporting One Health objectives by bridging the gap between human, animal, and environmental health. Their capacity to detect antimicrobial-resistant strains, monitor immune responses, and identify zoonotic pathogens at their source strengthens efforts to curb the global spread of AMR and emerging infectious diseases. As these technologies become increasingly affordable, multiplexed, and digitally integrated, their scalability will support widespread adoption, even in low- and middle-income countries where the burden of foodborne illness is often greatest.

In summary, biosensors represent a paradigm shift in the detection and management of *E. coli* within the poultry sector. Their continued development and strategic deployment will be central to advancing sustainable, safe, and resilient food systems that can meet the challenges of a rapidly growing global population and an increasingly interconnected world.

## Figures and Tables

**Table 1:** The strain and full name of various pathogenic *Escherichia coli* species along with the primary hosts, diseases associated, virulence factors, and their relevance to food safety.

Strain (Abbreviation)	Full Name	Primary Hosts	Disease(s) Caused	Key Virulence Factors	Notes on Relevance to Poultry/Food Safety
ETEC	Enterotoxigenic *E. coli*	Humans, animals	Traveler’s diarrhea, infant diarrhea	Heat-labile (LT) and heat-stable (ST) toxins	Rarely poultry-specific, but possible contamination in products
EPEC	Enteropathogenic *E. coli*	Humans (infants)	Infantile diarrhea	Attaching and effacing lesions, bundle-forming pilus (BFP)	Typically a human concern; not poultry-associated directly
EHEC	Enterohemorrhagic *E. coli*	Humans, cattle	Hemorrhagic colitis, HUS	Shiga toxins (Stx1, Stx2), intimin	Foodborne transmission; serious human pathogen via undercooked meat
EIEC	Enteroinvasive *E. coli*	Humans	Dysentery-like illness	Invasion plasmid antigens (ipa genes)	Human-specific; not associated with poultry
EAEC	Enteroaggregative *E. coli*	Humans	Persistent diarrhea (esp. in children)	Aggregative adherence fimbriae (AAF)	Human concern; not poultry-associated
APEC	Avian Pathogenic *E. coli*	Poultry	Colibacillosis (septicemia, airsacculitis, pericarditis, peritonitis)	*Iss*, *iucD*, *tsh*, *vat*, *papC*, *hlyF* genes	Major concern in poultry production and zoonotic potential
ESBL-EC	ESBL-producing *E. coli*	Poultry, humans	Various infections; antibiotic resistance	Extended-spectrum β-lactamases (e.g., CTX-M)	Emerging food safety threat; linked to antibiotic use in poultry

**Table 2:** Biosensor screening developments for *Escherichia coli* detection at different life stages in poultry production.

Life Stage	Biosensor Type	Target Biomarker	Sample Type	Linit of Detection (LOD)	Reference
In Ovo	Electrochemical DNA biosensor	Nucleic acid; *E. coli* O157:H7 antigen	Allantoic or amniotic fluid	2 × 10^−12^ mol/L DNA; 10 CFU/mL	Bhuiyan et al., 2025 [83] Zeinhom et al., 2018 [84]
Hatchlings	Microfluidic device with interdigitated electrodes	*E. coli* O157:H7 antigen	Tap water and waste water	3 bacterial cells/mL	Muhsin et al., 2022 [85]
Growing Birds	Label-free capacitive biosensor with Prussian blue/graphene oxide	*E. coli* somatic and capsular (O and K) antigen (LPS and bacterial wall)	Aerosol samples	8 bacterial cells/m3	Kumar et al., 2025 [87]
Grow-Out Phase	CRISPR-Cas-based nucleic acid sensor	Virulence-associated DNA sequences	Water, litter, cloacal swabs	Not provided	Gootenberg et al., 2017 [88]

**Table 3:** Implementation of biosensors for detection of antimicrobial resistance in *Escherichia coli* at different life stages in poultry production.

Life Stage	Target	Sample Type	Biosensor Approach	Limit of Detection (LOD)	Reference
Hatchling (1–7 days of age)	*E.coli* bacterial load, resistance	Cloacal swabs, environmental swabs	Colorimetric smartphone-based sensor	10^3^ to 10^9^ CFU/mL of *E. coli* O157:H7 was tested	Sun et al., 2019 [98]
Grow-out (2–6 weeks of age)	*E.coli*, AMR genes (e.g., *bla_TEM*)	Feed, feces, drinking water	SERS biosensors	Linear dependence on bacteria ranged from 10^1^ to 10^7^ CFU/mL (R^2^ = 0.9871); 1.5 CFU/mL (LOD)	Gao et al., 2017 [100]
Pre-slaughter (6+ weeks of age)	Antibiotic presence; *E. coli* antigens	Aerosol	Prussian blue/graphene oxide network on a screen-printed carbon electrode	5 to 8 bacterial cells/mL (LOD)	Kumar et al., 2025 [87]
Post-slaughter (meat products)	*E. coli* K12 antigen	Frozen chicken meat	Electrochemical impedance spectroscopy with anti-*E. coli* antibody (immunological)	10^3^ CFU/mL (LOD)	Helali et al., 2018 [103]

**Table 4:** Overview of biosensors targeting zoonotic *Escherichia coli*, including their sample matrices, detection limits, and turnaround times.

Biosensor Type	Target	Sample Type	Limit of Detection	Time	Reference
Label-free capacitive biosensor with Prussian blue/graphene oxide	*E. coli* somatic and capsular (O and K) antigen (LPS and bacterial wall)	Aerosol samples	8 bacterial cells/m^3^	Up to 30 min	Kumar et al., 2025 [87]
Microfluidic device with interdigitated electrodes	*E. coli* O157:H7 antigen	Environmental (water)	3 bacterial cells/mL	<2 h	Muhsin et al., 2022 [85]
Surface-Enhanced Raman Spectroscopy (SERS) Aptamer Biosensor	Whole *E. coli* cells (zoonotic strains)	Carcass rinse, wash water	~10^3^ CFU/mL	Minutes to ~1 h	Abuhelwa et al., 2024 [113]
Smartphone-integrated Colorimetric Biosensor	*E. coli* O157:H7 antigen	Milk	5.24 × 10^3^ CFU/mL	~30–60 min	Yang et al., 2021 [115]
